# Supplementary material for: Postmarketing active surveillance of myocarditis and pericarditis following vaccination with COVID-19 mRNA vaccines in persons aged 12 to 39 years in Italy: A multi-database, self-controlled case series study
Source: PLoS Med. 2022 Jul 28;19(7):e1004056. doi: 10.1371/journal.pmed.1004056 (PMC9333264; doi:10.1371/journal.pmed.1004056)
Supplement: S1 Table — (DOCX) [file pmed.1004056.s002.docx]

**Post-marketing active surveillance of myocarditis and pericarditis following vaccination with COVID-19 mRNA vaccines in persons aged 12-39 years in Italy: a multi-database, self-controlled case series study (Supporting information- S1 Table)**

**Table S1. Observational studies on safety of COVID-19 mRNA vaccines and myocarditis and/or pericarditis outcomes**

| **Author**  **Country** | **Population** | **Study period** | **Study design** | **Comparison** | **Risk period** | **Analysis** | **Outcome**  **Exposure**  **Results** | |
| --- | --- | --- | --- | --- | --- | --- | --- | --- |
| **Klein [1]**  US | n=10,162,227  ≥12 years | December 2020-June 2021 | Retrospective cohort study.  Active surveillance of data from health care provider participant sites (Kaiser Permanent ) | Rates in the risk period compared of individuals recently vaccinated with rate in comparisons period of individual less recently vaccinated | 0-21 days | Overall | **Myocarditis/Pericarditis**  Either mRNA vaccine  RR=1.18 (0.79-1.79) | |
|  |  |  |  |  | 0-21 days | 12-39 years | **Myocarditis/Pericarditis**  Either mRNA vaccine  Both doses RR=3.75 (1.38-12.84)  1 dose RR=3.67 (0.92-17.35)  2 dose RR=4.07 (1.45-14.18) | |
|  |  |  |  |  | 0-7 days |  | **Myocarditis/Pericarditis**  Either mRNA vaccine  Both doses RR=9.83 (3.35-35.77)  1 dose RR=7.27 (1.29-50.15)  2 dose RR=10.4 (3.54-37.76) | |
|  |  |  |  |  |  |  |  |  |
| **Barda [2]**  **Dagan [3]**  Israel | n=1,736,832  ≥16 years | December 2020-May 2021 | Retrospective cohort study data from Health care provider –Clalit Health Services | Rates in vaccinated in the risk period vs rates in unvaccinated individuals | 0-42 days | Overall | **Myocarditis**  BNT162b2  RR=3.24 (1.55-12.44) |  |
|  |  |  |  |  | 0-42 days | 16-39 years by sex | **Myocarditis**  BNT162b2  Females NA  Males RR=4.95(1.61-16.57) | |
|  |  |  |  |  |  |  | **Pericarditis**  BNT162b2  Females NA  Males RR=2.67(1.03-9.62) | |
|  |  |  |  |  |  | ≥ 40 years by sex | **Myocarditis**  BNT162b2  Females RR= 1.80 (0.00-4.73)  Males RR=1.20 (0.00-3.61) | |
|  |  |  |  |  |  |  | **Pericarditis**  BNT162b2  Females RR=0.67 (0.17-2.00)  Males RR=0.70 (0.15-3.58) | |
| **Patone [4]**  UK | n=38,615,491  ≥16 years | December 2020-August 2021 | SCCS | Self controlled | 1-7 days | Overall | **Myocarditis**  BNT162b2  1 dose IRR=1.45 (0.97-2.12)  2 dose IRR=1.75 (1.13-2.70) | **Myocarditis**  mRNA-1273  1 dose IRR=8.38 (3.53-19.91)  2 dose IRR=23.10 (6.46-82.56) |
|  |  |  |  |  |  |  | **Pericarditis**  BNT162b2  1 dose IRR=0.59 (0.32-1.07)  2 dose IRR=0.58 (0.33-1.04) | **Pericarditis**  mRNA-1273  1 dose NA  2 dose NA |
|  |  |  |  |  | 1-28 days | Overall | **Myocarditis**  BNT162b2  1 dose IRR=1.31 (1.03-1.66)  2 dose IRR=1.30 (0.98-1.72) | **Myocarditis**  mRNA-1273  1 dose IRR=2.97 (1.34-6.58)  2 dose IRR=9.48 (2.69-36.03) |
|  |  |  |  |  |  |  | **Pericarditis**  BNT162b2  Both doses No association | **Pericarditis**  mRNA-1273  Both doses No association |
|  |  |  |  |  | 1-28 days | By sex | **Myocarditis**  BNT162b2  Females 1 dose IRR=1.54 (1.08-2.20)  2 dose IRR=1.25 (0.81-2.20)  Males 1 dose IRR=1.16 (0.84-1.61)  2 dose IRR=1.39 (0.96-2.02) | **Myocarditis**  mRNA-1273  Females 1 dose NA  2 dose NA  Males 1 dose IRR=3.79 (1.59-9.04)  2 dose IRR=12.27 (2.77-54.37) |
|  |  |  |  |  |  |  | **Pericarditis**  BNT162b2  Females 1 dose IRR=0.84 (0.53-1.34)  2 dose IRR=0.80 (0.50-1.27)  Males 1 dose IRR=0.73 (0.51-1.04)  2 dose IRR=1.01 (0.73-1.38) | **Pericarditis**  mRNA-1273  Females 1 dose NA  2 dose NA  Males 1 dose IRR=1.96 (0.52-7.34)  2 dose NA |
|  |  |  |  |  |  | 16-39 years | **Myocarditis**  BNT162b2  1 dose IRR=1.83 (1.20-2.79)  2 dose IRR=3.40 (1.91-6.04) | **Myocarditis**  mRNA-1273  1 dose IRR=3.89 (1.60-9.44)  2 dose IRR=20.71 (4.02-106.68) |
|  |  |  |  |  |  |  | **Pericarditis**  BNT162b2  1 dose IRR=0.89 (0.51-1.54)  2 dose IRR=1.26 (0.62-2.54) | **Pericarditis**  mRNA-1273  1 dose IRR=2.49 (0.67-9.32)  2 dose NA |
|  |  |  |  |  |  |  |  |  |
| **Patone** **[6]**  UK | n=42,200,614  >13 years | December 2020-November 2021 | SCCS | Self controlled | 1-28 days | Overall | **Myocarditis**  BNT162b2  1 dose IRR=1.37 (1.12-1.67)  2 dose IRR=1.60 (1.31-1.97)  3 dose IRR=2.02 (1.40-2.91) | **Myocarditis**  mRNA-1273  1 dose IRR=1.80 (0.91-3.58)  2 dose IRR=13.71 (8.46-22.20)  3 dose NA |
|  |  |  |  |  |  | 16-39 years by sex | **Myocarditis**  BNT162b2  Females 1 dose IRR=1.44 (0.78-2.66)  2 dose IRR=1.37 (0.67-2.80)  3 dose NA  Males 1 dose IRR=1.66 (1.14-2.41)  2 dose IRR=3.41 (2.44-4.78)  3 dose IRR=7.60 (1.92-30.15) | **Myocarditis**  mRNA-1273  Females 1 dose IRR=2.88 (0.56-14.74)  2 dose IRR=7.55 (1.67-34.12)  3 dose NA  Males 1 dose IRR=2.34 (1.03-5.34)  2 dose IRR=16.52 (9.10-30.00)  3 dose NA |
|  |  |  |  |  |  |  |  |  |
| **Husby [7]**  Denmark | n=4 931 775  ≥12 years | October 2020 – October 2021 | Population based cohort study-  Nationwide Registries data linkage | Vaccinated vs unvaccinated | 28 days | Overall | **Myocarditis/Pericarditis**  BNT162b2  HR=1.34 (0.90-2.00) | **Myocarditis/Pericarditis**  mRNA-1273  HR=3.92 (2.30-6.68) |
|  |  |  |  |  |  | By sex | **Myocarditis/Pericarditis**  BNT162b2  Females HR=3.37 (1.82-7.65)  Males HR=0.82 (0.50-1.34) | **Myocarditis/Pericarditis**  mRNA-1273  Females HR=6.33 (2.11-18.96)  Males HR=3.22 (1.75-5.93) |
|  |  |  |  |  |  | 12-39 years | **Myocarditis/Pericarditis**  BNT162b2  HR=1.48 (0.74-2.98) | **Myocarditis/Pericarditis**  mRNA-1273  HR=5.24 (2.47-11.12) |

**References**

1. Klein NP, Lewis N, Goddard K, Fireman B, Zerbo O, Hanson KE, et al. Surveillance for adverse events after COVID-19 mRNA vaccination. *JAMA* 2021;326(14):1390–1399. doi:10.1001/jama.2021.15072

2. Barda N, Dagan N, Ben-Shlomo Y, Kepten E, Waxman J, Ohana R, et al. Safety of the BNT162b2 mRNA Covid- 34432976 19 vaccine in a nationwide setting. *N Engl J Med* 2021 Sep;385(12):1078-1090. doi: 10.1056/NEJMoa2110475

3. Dagan N, Barda N, Balicer RD. Adverse effects after BNT162b2 vaccine and SARS-CoV-2 infection, according to age and sex. *N Engl J Med* 2021 Dec;385(24):2299. doi:10.1056/NEJMc2115045

4. Patone M, Mei XW, Handunnetthi L, Dixon S, Zaccardi F, Shankar-Hari M, et al. Risks of myocarditis, pericarditis, and cardiac arrhythmias associated with COVID-19 vaccination or SARS-CoV-2 infection. *Nat Med* Dec*;*2021. doi:10.1038/s41591-021-01630-0

5. Patone M, Mei X W, Handunnetthi L, Zaccardi F, Shankar-Hari M, Watkinson P, et al. Risk of myocarditis following sequential COVID-19 vaccinations by age and sex. MedRxiv 2021.12.23.21268276 [Preprint]. 2021 [cited 2022 January 21]. Available from: https://www.medrxiv.org/content/10.1101/2021.12.23.21268276v1

6. Husby A, Hansen JV, Fosbøl E, Thiesson EM, Madsen M, Thomsen RW, et al. SARS-CoV-2 vaccination and myocarditis or myopericarditis: population based cohort study. *BMJ* 2021*;*375:e068665. doi:10.1136/bmj-2021-068665
