# Supplementary material for: Postmarketing active surveillance of myocarditis and pericarditis following vaccination with COVID-19 mRNA vaccines in persons aged 12 to 39 years in Italy: A multi-database, self-controlled case series study
Source: PLoS Med. 2022 Jul 28;19(7):e1004056. doi: 10.1371/journal.pmed.1004056 (PMC9333264; doi:10.1371/journal.pmed.1004056)
Supplement: S2 Table — n., number; yrs, years; COPD, chronic obstructive pulmonary disease; HIV, human immunodeficiency virus; NSAIDs, nonsteroidal anti-inflammatory drugs. (DOCX) [file pmed.1004056.s003.docx]

**Post-marketing active surveillance of myocarditis and pericarditis following vaccination with COVID-19 mRNA vaccines in persons aged 12-39 years in Italy: a multi-database, self-controlled case series study (Supporting information- S2 Table)**

**Table S2. Characteristics of mRNA vaccinated population aged 12-39 years (n. 2,861,809) from 27 December 2020 to 30 September 2021, by vaccine product.**

|  | **mRNA [n.(%)]** | **BNT162b2 [n.(%)]** | **mRNA-1273 [n.(%)]** |
| --- | --- | --- | --- |
| **Number of subjects** | 2,861,809 | 2,405,759 | 456,050 |
| **Sex** |  |  |  |
| Males | 1,458,703 (51.0%) | 1,214,517 (50.5%) | 244,186 (53.5%) |
| Females | 1,403,106 (49.0%) | 1,191,242 (49.5%) | 211,864 (46.5%) |
| **Charlson index** |  |  |  |
| ≥1 | 37,679 (1.3%) | 32,348 (1.3%) | 5,331 (1.2%) |
| **Hospitalizations in the last 2 years** |  |  |  |
| ≥1 | 294,859 (10.3%) | 246,487 (10.2%) | 47,372 (10.4%) |
| **Comorbidities** |  |  |  |
| COVID-19 diagnosis before vaccination | 238,667 (8.3%) | 198,081 (8.2%) | 40,586 (8.9%) |
| COPD | 163,804 (5.7%) | 139,260 (5.8%) | 24,544 (5.4%) |
| Chronic pulmonary disease | 10,405 (0.4%) | 8,718 (0.4%) | 1,687 (0.4%) |
| Chronic kidney failure | 5,789 (0.2%) | 4,941 (0.2%) | 848 (0.2%) |
| Neoplasms | 36,087 (1.3%) | 31,097 (1.3%) | 4,990 (1.1%) |
| Diabetes mellitus | 45,964 (1.6%) | 38,820 (1.6%) | 7,144 (1.6%) |
| Hematologic disease | 148,429 (5.2%) | 123,698 (5.1%) | 24,731 (5.4%) |
| Cardiovascular and cerebrovascular diseases | 52,539 (1.8%) | 44,837 (1.9%) | 7,702 (1.7%) |
| Hypertension | 48,023 (1.7%) | 40,625 (1.7%) | 7,398 (1.6%) |
| Hepatopathy | 9,669 (0.3%) | 7,931 (0.3%) | 1,738 (0.4%) |
| HIV | 5,736 (0.2%) | 4,404 (0.2%) | 1,332 (0.3%) |
| Rheumatic diseases | 25,906 (0.9%) | 22,243 (0.9%) | 3,663 (0.8%) |
| Cystic fibrosis | 1,192 (0.04%) | 1,101 (0.05%) | 91 (0.02%) |
| Neurological diseases | 116,248 (4.1%) | 97,748 (4.1%) | 18,500 (4.1%) |
| Peptic ulcer | 123,509 (4.3%) | 103,063 (4.3%) | 20,446 (4.5%) |
| Colitis | 11,249 (0.4%) | 9,588 (0.4%) | 1,661 (0.4%) |
| Celiac disease | 22,056 (0.8%) | 19,079 (0.9%) | 2,977 (0.7%) |
| Infection | 521,940 (18.2%) | 437,186 (18.2%) | 84,754 (18.6%) |
| **Prior drugs use** |  |  |  |
| Prescriptions in the last 12 months (1+) | 1,043,132 (36.5%) | 876,976 (36.5%) | 166,156 (36.4%) |
| Corticosteroids for systemic use | 100,096 (3.5%) | 84,220 (3.5%) | 15,876 (3.5%) |
| NSAIDs use | 26,108 (0.9%) | 21,621 (0.9%) | 4,487 (1.0%) |
| Estroprogestinics use | 27,783 (1.0%) | 23,282 (1.0%) | 4,501 (1.0%) |

n.: number; yrs: years; COPD: Chronic Obstructive Pulmonary Disease; HIV: Human Immunodeficiency Virus; NSAIDs: Non Steroidal Anti-Inflammatory Drugs
