# Supplementary material for: Postmarketing active surveillance of myocarditis and pericarditis following vaccination with COVID-19 mRNA vaccines in persons aged 12 to 39 years in Italy: A multi-database, self-controlled case series study
Source: PLoS Med. 2022 Jul 28;19(7):e1004056. doi: 10.1371/journal.pmed.1004056 (PMC9333264; doi:10.1371/journal.pmed.1004056)
Supplement: S3 Table — ATC, Anatomical Therapeutic Chemical Classification System; COPD, chronic obstructive pulmonary disease; HIV, human immunodeficiency virus; ICD, International Classification of Disease; NSAID, nonsteroidal anti-inflammatory drugs. (DOCX) [file pmed.1004056.s004.docx]

**Post-marketing active surveillance of myocarditis and pericarditis following vaccination with COVID-19 mRNA vaccines in persons aged 12-39 years in Italy: a multi-database, self-controlled case series study (Supporting information- S3 Table)**

**S3 Table. Definition of study comorbidities.**

| **Comorbidities** | **Databases** | | |
| --- | --- | --- | --- |
|  | **Hospital discharge** | **Pharmacy claims** | **Copayment exemption** |
|  | **ICD 9-CM codes**  **(in the last 5 years)** | **ATC codes**  **(in the last 12 months)** | **Exemption codes** |
| COPD | 490; 492; 493; 494; 496 | R03 | 057; 007 |
| Chronic pulmonary disease | 480-488; 491; 495; 518.81-518.84 | J05AH | 024 |
| Chronic kidney failure | 580; 582-585; 593; 753.12-753.14 |  | 023; 022; 061; 062 |
| Neoplasms | 140-209, V10 | L01 | 048 |
| Diabetes mellitus | 250 | A10 | 013 |
| Hematologic disease | 280-284; 285 (excl.285.1); 286-289 | B01AA; B01AB; B01AE; B01AF; B01AX; B02BD; B03 | 003 |
| Cardiovascular and cerebrovascular diseases | 390-398; 406-459 | B01AC; C01B; C01DA; C08DA; C08DB | 002; 021  0A02; 0B02; 0C02; 036 |
| Hypertension | 401-405 | C02; C03; C07; C08 (excl. C08DA; C08DB); C09 | 031; 0A31; 0031 |
| Hepatopathy | 456.0-456.2; 571-572; 573.0 |  | 008; 016 |
| Dementia /Alzheimer | 290; 294.1; 331.2 | N06DA, N06DX | 011; 029 |
| HIV | 042 | J05AE; J05AF; J05AG; J05AR | 020 |
| Rheumatic diseases | 446.5; 710; 714; 720; 725; 696 | L04 | 006;028; 030; 045; 054; 067 |
| Cystic fibrosis | 277.0 | R07AX | 018 |
| Neurological diseases | 296.3; 238.7; 311; 332; 340; 345; 348.39 | N03A; N04B; N05A; N06A | 017; 038; 044; 046 |
| Peptic Ulcer | 531-533 | A02B |  |
| Colitis | 555; 556 |  | 009 |
| Celiac disease | 579.0 |  | 059 |
| Infection (in the last 12 months) | 053; 599.0; 010-018; 031; 078.5; 052-054; 136.3; 117.5 | J01; J02; J04; J05 (excl. J05AE; J05AF; J05AG; J05AR; J05AH) | 055 |
| Corticosteroids for systemic use |  | H02A |  |
| NSAIDs use |  | M01A |  |
| Estroprogestinics use |  | G03 |  |

ICD: International Classification of Disease; ATC: Anatomical Therapeutic Chemical Classification System; COPD: Chronic obstructive pulmonary disease; HIV: Human Immunodeficiency Virus; NSAIDs: Non-steroidal anti-inflammatory drugs
