# Supplementary material for: Postmarketing active surveillance of myocarditis and pericarditis following vaccination with COVID-19 mRNA vaccines in persons aged 12 to 39 years in Italy: A multi-database, self-controlled case series study
Source: PLoS Med. 2022 Jul 28;19(7):e1004056. doi: 10.1371/journal.pmed.1004056 (PMC9333264; doi:10.1371/journal.pmed.1004056)
Supplement: S4 Table — CI, confidence interval; n., number; Ref., reference period (unexposed period); RI, relative incidence; SCCS, self-controlled cases series. (DOCX) [file pmed.1004056.s005.docx]

**Post-marketing active surveillance of myocarditis and pericarditis following vaccination with COVID-19 mRNA vaccines in persons aged 12-39 years in Italy: a multi-database, self-controlled case series study (Supporting information- S4 Table)**

**S4 Table. Relative incidence estimated by SCCS by vaccine product and risk intervals: 346 myocarditis/pericarditis events in the BNT162b2 and 95 events in the mRNA-1273 vaccinated population aged 12-39 years from 27 December 2020 to 30 September 2021.**

| **Risk interval** | **Dose**​ | **BNT162b2 (n. 346)** | | **mRNA-1273 (n. 95)** | |
| --- | --- | --- | --- | --- | --- |
|  |  | **Events in the risk interval (n)** | **Relative Incidence**  **(95% CI)** | **Events in the risk interval (n)** | **Relative Incidence**  **(95% CI)** |
| [0-21)​ | Dose 1​ | 35 | 1.06 (0.68-1.67) | 15 | 1.60 (0.66-3.90) |
| ​ | Dose 2​ | 39 | 2.01 (1.27-3.19) | 25 | 2.27 (1.01-5.09) |
| [0-7)​ | Dose 1​ | 14 | 1.22 (0.67-2.25) | 11 | 4.33 (1.71-10.98) |
| ​ | Dose 2​ | 22 | 3.35 (1.96-5.72) | 23 | 6.20 (2.69-14.28) |
| [7-14)​ | Dose 1​ | 10 | 0.89 (0.45-1.79) | 3 | 1.18 (0.32-4.37) |
| ​ | Dose 2​ | 7 | 1.07 (0.49-2.34) | 0 | - |
| [14-21)​ | Dose 1​ | 11 | 1.07 (0.54-2.10) | 1 | 0.40 (0.05-3.40) |
| ​ | Dose 2​ | 10 | 1.61 (0.77-3.34) | 2 | 0.60 (0.14-2.62) |
| *Ref.* ​ | *​* | *272* | *1* | *55* | *1* |

SCCS: Self-Controlled Cases Series; n.: number; CI: Confidence interval; Ref.: reference period (unexposed period)
