# Supplementary material for: Postmarketing active surveillance of myocarditis and pericarditis following vaccination with COVID-19 mRNA vaccines in persons aged 12 to 39 years in Italy: A multi-database, self-controlled case series study
Source: PLoS Med. 2022 Jul 28;19(7):e1004056. doi: 10.1371/journal.pmed.1004056 (PMC9333264; doi:10.1371/journal.pmed.1004056)
Supplement: S5 Table — *Considering the small number of cases, it was not possible to provide any estimates. CI, confidence interval; n., number; Ref., reference period (unexposed baseline period); RI, relative incidence. (DOCX) [file pmed.1004056.s006.docx]

**Post-marketing active surveillance of myocarditis and pericarditis following vaccination with COVID-19 mRNA vaccines in persons aged 12-39 years in Italy: a multi-database, self-controlled case series study (Supporting information- S5 Table)**

**S5 Table. Relative incidence estimated by SCCS in the [0-7) risk period after mRNA vaccination in the vaccinated population aged 12-39 years from 27 December 2020 to 30 September 2021 by sex, age group and vaccine product.**

| **Age, years** | **Sex** | **Risk interval** | **Dose**​ | **BNT162b2 (n. 346)** | | | **mRNA-1273 (n. 95)** | | |
| --- | --- | --- | --- | --- | --- | --- | --- | --- | --- |
|  |  |  |  | **Events in the risk interval (n)** | **Relative Incidence**  **(95% CI)** | **Events in the risk interval (n)** | | **Relative Incidence**  **(95% CI)** |  |
| **12-39** | **Males+Females** | **[0-7)** | Dose 1 | 14 | 1.22 (0.67-2.25) | 11 | | 4.33 (1.71-10.98) |  |
|  |  |  | Dose 2 | 22 | 3.35 (1.96-5.72) | 23 | | 6.20 (2.69-14.28) |  |
|  |  | *Ref.* |  | *272* | *1* | *55* | | *1* |  |
|  | **Males** | **[0-7)** | Dose 1 | 9 | 1.38 (0.62-3.04) | 10 | | 8.78 (2.53-30.51) |  |
|  |  |  | Dose 2 | 13 | 3.05 (1.53-6.10) | 19 | | 9.72 (3.16-29.89) |  |
|  |  | *Ref.* |  | *184* | *1* | *38* | | *1* |  |
|  | **Females** | **[0-7)** | Dose 1 | 5 | 0.94 (0.36-2.46) | 1 | | 0.69 (0.08-5.75) |  |
|  |  |  | Dose 2 | 9 | 3.81 (1.66-8.72) | 4 | | 2.08 (0.45-9.72) |  |
|  |  | *Ref.* |  | *88* | *1* | *17* | | *1* |  |
|  |  |  |  |  |  |  | |  |  |
| **12-17** | **Males+Females** | **[0-7)** | Dose 1 | 3 | 0.71 (0.13-3.82) | 0 | | * |  |
|  |  |  | Dose 2 | 7 | 4.06 (1.11-14.82) | 3 | | * |  |
|  |  | *Ref.* |  | 31 | *1* | 7 | | *1* |  |
| **18-29** | **Males+Females** | **[0-7)** | Dose 1 | 7 | 1.50 (0.66-3.40) | 9 | | 5.39 (1.77-16.40) |  |
|  |  |  | Dose 2 | 11 | 3.92 /1.78-8.66) | 18 | | 8.16 (2.82-23.62) |  |
|  |  | *Ref.* |  | *121* | *1* | *28* | | *1* |  |
| **30-39** | **Males+Females** | **[0-7)** | Dose 1 | 4 | 1.12 (0.40-3.14) | 2 | | 3.55 (0.58-21.69) |  |
|  |  |  | Dose 2 | 4 | 1.88 (0.65-5.33) | 2 | | 1.97 (0.32-11.93) |  |
|  |  | **[7-14)** | Dose 1 | 4 | 1.20 (0.42-3.46) | 2 | | 3.55 (0.64-19.73) |  |
|  |  | *Ref.* |  | *120* | *1* | *20* | | *1* |  |

*considering the small number of cases, it was not possible to provide any estimates

n.: number; CI: Confidence Interval; Ref.: reference period (unexposed baseline period)
