# Supplementary material for: Postmarketing active surveillance of myocarditis and pericarditis following vaccination with COVID-19 mRNA vaccines in persons aged 12 to 39 years in Italy: A multi-database, self-controlled case series study
Source: PLoS Med. 2022 Jul 28;19(7):e1004056. doi: 10.1371/journal.pmed.1004056 (PMC9333264; doi:10.1371/journal.pmed.1004056)
Supplement: S6 Table — *Adjusted by calendar period. **Excess cases are not given when the 95% CI of RI included the null effect. CI, confidence interval; n., number; Ref., reference period (unexposed period); RI, relative incidence; SCCS, self-controlled cases series. (DOCX) [file pmed.1004056.s007.docx]

**Post-marketing active surveillance of myocarditis and pericarditis following vaccination with COVID-19 mRNA vaccines in persons aged 12-39 years in Italy: a multi-database, self-controlled case series study (Supporting information- S6 Table)**

**S6 Table. Adjusted relative incidence estimated by SCCS and excess cases per 100,000 vaccinated by risk intervals: 232 myocarditis and/or pericarditis events in the BNT162b2 vaccinated males aged 12-39 years from 27 December 2020 to 30 September 2021.**

| **Risk interval** | **Dose**​ | **Events in the risk interval (n)** | **Adjusted Relative Incidence (95% CI)*** | **Excess cases per 100,000 vaccinated (95% CI)**** |
| --- | --- | --- | --- | --- |
| [0-7)​ | Dose 1​ | 9 | 1.53 (0.71-3.31) |  |
| ​ | Dose 2​ | 13 | 3.45 (1.78-6.68) | 1.0 (0.3-1.8) |
| [7-14)​ | Dose 1​ | 9 | 1.53 (0.74-3.17) |  |
| ​ | Dose 2​ | 4 | 1.01 (0.37-2.75) |  |
| [14-21)​ | Dose 1​ | 8 | 1.41 (0.63-3.14) |  |
| ​ | Dose 2​ | 5 | 1.29 (0.49-3.42) |  |
| *Ref.* ​ | ​ | *184* | *1.0* |  |

SCCS: Self-Controlled Cases Series; n.: number; CI: Confidence interval; Ref.: reference period (unexposed period). *adjusted by calendar period; **excess cases are not given when the 95% CI of RI included the null effect
