# Supplementary material for: Postmarketing active surveillance of myocarditis and pericarditis following vaccination with COVID-19 mRNA vaccines in persons aged 12 to 39 years in Italy: A multi-database, self-controlled case series study
Source: PLoS Med. 2022 Jul 28;19(7):e1004056. doi: 10.1371/journal.pmed.1004056 (PMC9333264; doi:10.1371/journal.pmed.1004056)
Supplement: S11 Table — *Adjusted by calendar period. **Excess cases are not given when the 95% CI of RI included the null effect. CI, confidence interval; n., number; Ref., reference period (unexposed period); RI, relative incidence; SCCS, self-controlled cases series. (DOCX) [file pmed.1004056.s012.docx]

**Post-marketing active surveillance of myocarditis and pericarditis following vaccination with COVID-19 mRNA vaccines in persons aged 12-39 years in Italy: a multi-database, self-controlled case series study (Supporting information- S11 Table)**

**S11 Table. Adjusted relative incidence estimated by SCCS and excess cases per 100,000 vaccinated by risk intervals: 70 myocarditis and/or pericarditis events in the mRNA-1273 vaccinated males aged 12-39 years from 27 December 2020 to 30 September 2021.**

| **Risk interval** | **Dose**​ | **Events in the risk interval (n)** | **Adjusted Relative Incidence (95% CI)*** | **Excess cases per 100,000 vaccinated (95% CI)**** |
| --- | --- | --- | --- | --- |
| [0-7)​ | Dose 1​ | 10 | 12.28 (4.09-36.83) | 3.8 (1.5-6.3) |
| ​ | Dose 2​ | 19 | 11.91 (3.88-36.53) | 8.8 (4.9-12.9) |
| [7-14)​ | Dose 1​ | 2 | 2.19 (0.42-11.41) |  |
| ​ | Dose 2​ | 0 | — |  |
| [14-21)​ | Dose 1​ | 0 | — |  |
| ​ | Dose 2​ | 1 | 0.65 (0.08-5.37) |  |
| *Ref.* ​ | ​ | *38* | *1.0* |  |

SCCS: Self-Controlled Cases Series; n.: number; CI: Confidence interval; Ref.: reference period (unexposed period). *adjusted by calendar period; **excess cases are not given when the 95% CI of RI included the null effect
