# Supplementary material for: Postmarketing active surveillance of myocarditis and pericarditis following vaccination with COVID-19 mRNA vaccines in persons aged 12 to 39 years in Italy: A multi-database, self-controlled case series study
Source: PLoS Med. 2022 Jul 28;19(7):e1004056. doi: 10.1371/journal.pmed.1004056 (PMC9333264; doi:10.1371/journal.pmed.1004056)
Supplement: S15 Table — *Adjusted by calendar period. **Excess cases are not given when the 95% CI of RI included the null effect. CI, confidence interval; n., number; Ref., reference period (unexposed period); RI, relative incidence; SCCS, self-controlled cases series. (DOCX) [file pmed.1004056.s016.docx]

**Post-marketing active surveillance of myocarditis and pericarditis following vaccination with COVID-19 mRNA vaccines in persons aged 12-39 years in Italy: a multi-database, self-controlled case series study (Supporting information- S15 Table)**

**S15 Table. Adjusted relative incidence estimated by SCCS and excess cases per 100,000 vaccinated by risk intervals: 27 myocarditis and/or pericarditis events in the mRNA-1273 vaccinated population aged 30-39 years from 27 December 2020 to 30 September 2021.**

| **Risk interval** | **Dose**​ | **Events in the risk interval (n)** | **Adjusted Relative Incidence (95% CI)*** | **Excess cases per 100,000 vaccinated (95% CI)**** |
| --- | --- | --- | --- | --- |
| [0-7)​ | Dose 1 | 2 | 6.57 (1.32-32.63) | 1.0 (^NE-3.3) |
| ​ | Dose 2 | 2 | 3.22 (0.69-15.10) |  |
| [7-14)​ | Dose 1 | 2 | 5.87 (1.34-25.74) | 1.0 (^NE-3.3) |
| ​ | Dose 2 | 0 | — |  |
| [14-21)​ | Dose 1 | 1 | 2.56 (0.23-28.57) |  |
| ​ | Dose 2 | 0 | — |  |
| *Ref.* ​ |  | *20* | *1.0* |  |

SCCS: Self-Controlled Cases Series; n.: number; CI: Confidence interval; Ref.: reference period (unexposed period); NE: Not estimable. *adjusted by calendar period; **excess cases are not given when the 95% CI of RI included the null effect; ^Considering the small number of cases, bootstrapping produced implausible results for inferior limit of 95% CIs.
