# Supplementary material for: Postmarketing active surveillance of myocarditis and pericarditis following vaccination with COVID-19 mRNA vaccines in persons aged 12 to 39 years in Italy: A multi-database, self-controlled case series study
Source: PLoS Med. 2022 Jul 28;19(7):e1004056. doi: 10.1371/journal.pmed.1004056 (PMC9333264; doi:10.1371/journal.pmed.1004056)
Supplement: S16 Table — *Adjusted by calendar period. **Only participants vaccinated and with event in each period were included in this analysis. ***Considering the small number of cases, it was not possible to provide any estimates. CI, confidence interval; SCCS, self-controlled cases series. (DOCX) [file pmed.1004056.s017.docx]

**Post-marketing active surveillance of myocarditis and pericarditis following vaccination with COVID-19 mRNA vaccines in persons aged 12-39 years in Italy: a multi-database, self-controlled case series study (Supporting information- S16 Table)**

**S16 Table. Sensitivity analyses.**

|  | **Risk**  **interval** | **Dose** | **Adjusted**  **Relative Incidence (95% CI)*** | | |
| --- | --- | --- | --- | --- | --- |
|  |  |  | **BNT162b2** | **mRNA-1273** |  |
| **Reference analysis (SCCS modified for event-dependent exposures)** | **[0-7)** | **Dose 1** | **1.27 (0.70-2.31)** | **6.55 (2.73-15.72)** |  |
|  |  | **Dose 2** | **3.39 (2.02-5.68)** | **7.59 (3.26-17.65)** |  |
| **a) Observation time period (I)**  analysis restricted to the study period from 27 December 2020 to 31 May 2021 (n. 85)** | **[0-7)** | Dose 1 | 0.48 (0.10-2.20) | *** |  |
|  |  | Dose 2 | 3.29 (1.22-8.88) | *** |  |
| **a) Observation time period (II)**  analysis restricted to the study period from 1 June 2021 to 30 September 2021 (n. 160)** | **[0-7)** | Dose 1 | 2.05 (0.97-4.33) | 6.26 (2.15-15.22) |  |
|  |  | Dose 2 | 4.32 (2.11-8.83) | 13.43 (4.65-38.76) |  |
| **a) Exposure time period (I)**  excluding day 0 from [0-7) day risk interval (n. 441) | **[0-7)** | Dose 1 | 1.39 (0.75-2.55) | 7.68 (3.21-18.39) |  |
|  |  | Dose 2 | 3.96 (2.36-6.62) | 8.76 (3.78-20.29) |  |
| **a) Exposure time period (II)**  extending the exposure period to 0-28 days (n. 441) | **[0-7)** | Dose 1 | 1.29 (0.71-2.33) | 5.97 (2.38-14.96) |  |
|  |  | Dose 2 | 3.08 (1.82-5.24) | 7.49 (2.89-19.42) |  |
| **a) Exposure time period (III)**  reducing the exposure period to 0-14 days (n. 441) | **[0-7)** | Dose 1 | 1.21 (0.67-2.19) | 7.43 (3.27-16.88) |  |
|  |  | Dose 2 | 3.18 (1.91-5.28) | 8.00 (3.58-17.89) |  |
| **b) Heterologous vaccination (I)**  excluding individuals with two different products in the first and second dose (n. 440) | **[0-7)** | Dose 1 | 1.27 (0.69-2.31) | 6.56 (2.73-15.74) |  |
|  |  | Dose 2 | 3.39 (2.02-5.68) | 7.59 (3.26-17.66) |  |
| **b) Heterologous vaccination (II)**  censoring 1 subjects who received a different product at the II dose (from mRNA-1273 to BNT162b2, n. 441) | **[0-7)** | Dose 1 | 1.27 (0.69-2.31) | 6.61 (2.75-18.90) |  |
|  |  | Dose 2 | 3.39 (2.02-5.68) | 7.61 (3.27-17.72) |  |
| **c) SARS-CoV-2 infection**  restricting the analyses to subjects without a SARS-CoV-2 positive test before and during the study period (n. 378) | **[0-7)** | Dose 1 | 1.17 (0.64-2.25) | 5.66 (1.99-16.13) |  |
|  |  | Dose 2 | 3.13 (1.82-5.39) | 7.85 (3.26.18.94) |  |

SCCS: Self-Controlled Cases Series; CI: Confidence interval. *adjusted by calendar period; **only subjects vaccinated and with event in each period were included in this analysis ***considering the small number of cases, it was not possible to provide any estimates
