# Supplementary material for: Postmarketing active surveillance of myocarditis and pericarditis following vaccination with COVID-19 mRNA vaccines in persons aged 12 to 39 years in Italy: A multi-database, self-controlled case series study
Source: PLoS Med. 2022 Jul 28;19(7):e1004056. doi: 10.1371/journal.pmed.1004056 (PMC9333264; doi:10.1371/journal.pmed.1004056)
Supplement: S17 Table — *Adjusted by calendar period. CI, confidence interval; SCCS, self-controlled cases series. (DOCX) [file pmed.1004056.s018.docx]

**Post-marketing active surveillance of myocarditis and pericarditis following vaccination with COVID-19 mRNA vaccines in persons aged 12-39 years in Italy: a multi-database, self-controlled case series study (Supporting information- S17 Table)**

**S17 Table. Sensitivity analyses: standard SCCS method.**

| **0-21 risk period** | **Risk**  **interval** | **Dose** | **Adjusted**  **Relative Incidence (95% CI)*** | |
| --- | --- | --- | --- | --- |
|  |  |  | **BNT162b2** | **mRNA-1273** |
| **Reference analysis (SCCS modified for event-dependent exposures)** | **[0-7)** | **Dose 1** | **1.27 (0.70-2.31)** | **6.55 (2.73-15.72)** |
|  |  | **Dose 2** | **3.39 (2.02-5.68)** | **7.59 (3.26-17.65)** |
| **d) standard SCCS beginning observation at exposure**  starting the observation time at the first (n. 211) and second dose (n. 130) | [0-7) | Dose 1 | 1.27 (0.69-2.33) | 5.59 (2.37-13.17) |
|  |  | Dose 2 | 3.35 (1.98-5.69) | 6.20 (2.45-15.68) |
| **e) standard SCCS beginning observation at time 0**  starting the observation time at 0 (n. 441) | [0-7) | Dose 1 | 1.79 (1.04-3.08) | 6.70 (3.29-13.64) |
|  |  | Dose 2 | 3.90 (2.47-6.16) | 18.25 (9.73-34.21) |
| **f) standard SCCS with pre-risk period**  starting the observation time at 0 with [-28,-1] for dose 1 pre-risk period (n. 441) | [0-7) | Dose 1 | 1.62 (0.94-2.80) | 5.79 (2.79-12.03) |
|  |  | Dose 2 | 3.61(2.29-5.71) | 16.54 (8.80-31-08) |
| **f) standard SCCS with pre-risk period**  starting the observation time at 0 with [-28,-1] for dose 1 and [-14,-1] for dose 2 pre-risk period (n. 441) | [0-7) | Dose 1 | 1.59 (0.92-2.73) | 5.47 (2.63-11.41) |
|  |  | Dose 2 | 3.47 (2.19-5.49) | 15.05 (7.89-28.69) |
| **f) standard SCCS with pre-risk period**  starting the observation time at 0 with [-28,-1] for both doses pre-risk period (n. 441) | [0-7) | Dose 1 | 2.23 (1.26-3.92) | 7.24 (3.40-15.44) |
|  |  | Dose 2 | 3.28 (2.07–5.20) | 13.24 (6.92-25.35) |
| **g) standard SCCS removing post event exposure**  removing post event Dose 1 and Dose 2 (n. 441) | [0-7) | Dose 1 | 5.89 (3.32-10.44) | model did not converge |
|  |  | Dose 2 | 17.44 (10.41-29.19) |  |

SCCS: Self-Controlled Cases Series; CI: Confidence interval. *adjusted by calendar period
