# Supplementary material for: Postmarketing active surveillance of myocarditis and pericarditis following vaccination with COVID-19 mRNA vaccines in persons aged 12 to 39 years in Italy: A multi-database, self-controlled case series study
Source: PLoS Med. 2022 Jul 28;19(7):e1004056. doi: 10.1371/journal.pmed.1004056 (PMC9333264; doi:10.1371/journal.pmed.1004056)
Supplement: S18 Table — *Adjusted by calendar period. **Excess cases are not given when the 95% CI of RI included the null effect. CI, confidence interval; n., number; Ref., reference period (unexposed period); RI, relative incidence; SCCS, self-controlled cases series. (DOCX) [file pmed.1004056.s019.docx]

**Post-marketing active surveillance of myocarditis and pericarditis following vaccination with COVID-19 mRNA vaccines in persons aged 12-39 years in Italy: a multi-database, self-controlled case series study (Supporting information- S18 Table)**

**S18 Table. Adjusted relative incidence estimated by SCCS and excess cases per 100,000 vaccinated by risk intervals: 1,759 myocarditis and/or pericarditis events in the BNT162b2 and 291 events in the mRNA-1273 vaccinated population aged ≥40 years from 27 December 2020 to 30 September 2021 (ancillary analysis).**

| **Sex** | **Risk**  **interval** | **Dose** | **BNT162b2**  **Vaccinated 6,835,634 (n. cases 1,759)** | | |  | **mRNA-1273**  **Vaccinated 1,041,582 (n. cases 291)** | | |
| --- | --- | --- | --- | --- | --- | --- | --- | --- | --- |
|  |  |  | **Events in the risk interval (n)** | **Adjusted**  **Relative Incidence (95% CI)*** | **Excess**  **cases per**  **100,000**  **Vaccinated (95% CI)**** |  | **Events in the risk interval (n)** | **Adjusted**  **Relative Incidence (95% CI)*** | **Excess**  **cases per**  **100,000**  **Vaccinated (95% CI)**** |
| **Males+Females** | **[0-7)** | Dose 1 | 39 | 0.59 (0.42-0.82) |  |  | 7 | 0.56 (0.23-1.36) |  |
|  |  | Dose 2 | 43 | 0.84 (0.61-1.16) |  |  | 10 | 1.11 (0.57-2.17) |  |
|  |  | *Ref.* | *1,479* | *1* |  |  | *247* | *1* |  |
| **Males** | **[0-7)** | Dose 1 | 16 | 0.43 (0.26-0.71) |  |  | 1 | 0.12 (0.01-1.31) |  |
|  |  | Dose 2 | 23 | 0.85 (0.54-1.33) |  |  | 6 | 1.11 (0.47-2.66) |  |
|  |  | *Ref.* | *824* | *1* |  |  | *134* | *1* |  |
| **Females** | **[0-7)** | Dose 1 | 23 | 0.76 (0.49-1.19) |  |  | 6 | 1.16 (0.46-2.92) |  |
|  |  | Dose 2 | 20 | 0.84 (0.53-1.33) |  |  | 4 | 1.11 (0.39-3.13) |  |
|  |  | *Ref.* | *655* | *1* |  |  | *113* | *1* |  |

SCCS: Self-Controlled Cases Series; n.: number; CI: Confidence interval; Ref.: reference period (unexposed period). *adjusted by calendar period; **excess cases are not given when the 95% CI of RI included the null effect
