# Supplementary material for: Postmarketing active surveillance of myocarditis and pericarditis following vaccination with COVID-19 mRNA vaccines in persons aged 12 to 39 years in Italy: A multi-database, self-controlled case series study
Source: PLoS Med. 2022 Jul 28;19(7):e1004056. doi: 10.1371/journal.pmed.1004056 (PMC9333264; doi:10.1371/journal.pmed.1004056)
Supplement: S1 Fig — CDM, common data model. (DOCX) [file pmed.1004056.s020.docx]

**Post-marketing active surveillance of myocarditis and pericarditis following vaccination with COVID-19 mRNA vaccines in persons aged 12-39 years in Italy: a multi-database, self-controlled case series study (Supporting information- S1 Figure)**

**S1 Figure. Diagram showing the data flow when using *TheShinISS* to locally process health care data structured according to a Common Data Model.**

CDM: Common Data Model
