# Supplementary material for: Postmarketing active surveillance of myocarditis and pericarditis following vaccination with COVID-19 mRNA vaccines in persons aged 12 to 39 years in Italy: A multi-database, self-controlled case series study
Source: PLoS Med. 2022 Jul 28;19(7):e1004056. doi: 10.1371/journal.pmed.1004056 (PMC9333264; doi:10.1371/journal.pmed.1004056)
Supplement: S4 Fig — (*) Considering the small number of cases in the vaccinated with mRNA-1273 of age 12–17 years, it was not possible to provide any estimates. CI, confidence interval; F, females; M, males; RI, relative incidence. (DOCX) [file pmed.1004056.s023.docx]

**Post-marketing active surveillance of myocarditis and pericarditis following vaccination with COVID-19 mRNA vaccines in persons aged 12-39 years in Italy: a multi-database, self-controlled case series study (Supporting information- S4 Figure)**

**S4 Figure. Adjusted relative incidence in the [0-7) risk period after mRNA vaccination in the vaccinated population aged 12-39 years from 27 December 2020 to 30 September 2021 by vaccine product, sex and age group.**

RI: Relative Incidence; CI: Confidence Interval; M: Males; F: Females. (*) Considering the small number of cases, it was not possible to provide any estimates
