# Supplementary material for: Postmarketing active surveillance of myocarditis and pericarditis following vaccination with COVID-19 mRNA vaccines in persons aged 12 to 39 years in Italy: A multi-database, self-controlled case series study
Source: PLoS Med. 2022 Jul 28;19(7):e1004056. doi: 10.1371/journal.pmed.1004056 (PMC9333264; doi:10.1371/journal.pmed.1004056)
Supplement: Attachment — Submitted filename: massari.pdf [file pmed.1004056.s024.pdf]

This paper presents the analysis of routinely collected data on specific side effects of mRNA vaccines in young people in Italy.

I was asked for a statistical report and I interpret that to include all aspects of the design and conduct of the study.

Points of detail

Page 7 I wonder whether the fact that all four regions are in the north of Italy is worth mentioning. I have no idea about north–south health gradients in Italy but I imagine there must be some.

Page 7 I think in the results we should be told how many people had to be excluded for missing sex, age, or vaccine details. I imagine this was quite rare but unless we know we cannot say whether any extra analysis is needed to cope with missingness.

Page 13 on onwards The authors defined the period of interest as 0-21 days (see page 7) but their results and analysis focus on the three separate time periods. I can see that the hypothesis may have been that early effects were to be expected or that the patho–physiology demands the sub–division but why not present the 0–21 results? After all as a patient I want to know if I am going to experience a side effect and am not really interested in whether it occurs on day 5, 10 or 15.

Page 14 Perhaps have another row with the overall totals (346 and 95)? It took me a moment to work out why $11 + 23 + 3 + 1 + 2 \neq 55$

Page 14 The subset analyses were pre–specified but presenting them does lead the reader to assume that the differences beween men and women and between age groups are substantial. Was any form of formal interaction test carried out? If not then I think it would be a helpful addition.

Page 19 Would it be helpful to tabulate the results of the previous studies in some way to ease comparison of the totality of global evidence?

Page 26 I do not think elaborate is quite the right word here. As an adjective it usually means complicated. Perhaps produced or compiled might be better.

Figures Suddenly introducing the trade names seems strange. Perhap cross–reference them to the names used in the main text? Articles about vaccination may attract lay readers to an open access journal and, at least in my country, the lay public will not be familiar with them. In

> fact I think the only place I have seen Comirnaty is on my vaccine pass (which incidentally tells me it is manufactured by Biontech just to confuse matters further). I did not know the name Spikevax at all.

Nice to see the R package used properly cited.

Points of more substance

There is much vaccine hesitancy in the world and this article has a focus, as planned, on side effects. The authors do refer to the balance between side effects and benefits but I feel that they rather understate this. I know their research has not studied the benefits but a slightly stronger comment on benefits versus side effects would not be out of place.

Summary

No major issues.

Michael Dewey
