# Supplementary material for: Postmarketing active surveillance of myocarditis and pericarditis following vaccination with COVID-19 mRNA vaccines in persons aged 12 to 39 years in Italy: A multi-database, self-controlled case series study
Source: PLoS Med. 2022 Jul 28;19(7):e1004056. doi: 10.1371/journal.pmed.1004056 (PMC9333264; doi:10.1371/journal.pmed.1004056)
Supplement: Attachment — Submitted filename: Massari_ResponsEditor PLoSM (20.06.2022).pdf [file pmed.1004056.s027.pdf]

Post-marketing active surveillance of myocarditis and pericarditis following vaccination with COVID-19 mRNA vaccines in persons aged 12-39 years in Italy: a multi-database, self-controlled case series study

Massari M & Spila Alegiani S et al.

Remaining issue to be addressed (lines and pages are referred to the clean version of the manuscript)

---

1) The Author Summary should immediately follow the Abstract in your revised manuscript.
The Author Summary has been prepared and it follows the Abstract in the revised manuscript.

2) Lines 60-61 'The main study limitations were that the outcome was not validated through review of clinical records, and there was an absence of information on the length of hospitalization and, thus, the severity of the outcome.'
The phrase was updated as requested see lines 59-61 page 3.

3) Line 163: Please specify the variables that were considered relevant.
The variables have been specified, see lines 189-190 page 8.

4) Line 195: Please update to 'we extended the exposure period to 28 days as well reducing it to 14 days.'
We have updated the phrase as suggested, see line 222 page 9.

5) Line 200: Please describe these further sensitivity analyses.
The further sensitivity analyses have been described in the method section, see lines 227-229 page 9-10 (we have added the letter sequence, d-g, of the sensitivity analyses in S17 table).

6) Line 204: "Please update to 'This study is reported as per the REporting of studies Conducted using Observational Routinely-collected Data for Pharmacoepidemiology (RECORD-PE) checklist (S1 Checklist).'
We have updated as suggested, see lines 232-233 page 10.

7) Please update your RECORD-PE checklist to use section names and paragraph numbers rather than page/line numbers (these will either change or not be present in the final published version).
We have updated the RECORD-PE checklist with section names and paragraph numbers.

8) Line 215 Please include the actual number of missing observations as well as the proportion.
We have included actual number of missing information, see lines 244-245 page 10.

9) Line 269: Please update to 'Of note, there were…'
We have updated as suggested, see line 298 page 15.

10) Line 280-281: Please include the 95% Cis
We have included 95% CIs as requested, see line 309 page 15. Please note that it was only possible to estimate the upper limit of the CI.

11) Line 386: “Please update to ‘It is reassuring that the sensitivity analysis (S17 Table) in which the observation time began at first or second vaccination dose in a standard SCCS method gave similar estimates to our main analysis.’
We have updated as suggested, see lines 414-416 page 20.

12) Line 391: ‘Such bias is probably minimal’ – please either provide a reference to support this claim or remove it
We have removed it as suggested, see line 419 page 20.

13) For internet references (e.g. reference 8) please include the data accessed
We have included the data accessed in all the internet references.

14) Please ensure all Supporting Information items (e.g. Supporting Tables/Figures) are cited in the main text.
We have checked that all supporting information are cited in the main text and we confirm that were cited properly in the text. We have added a sentence (“unadjusted estimated in S5 Table”) at lines 285 at page 14 to specify that S5 table was referred to unadjusted estimates.
