# Supplementary material for: Postmarketing active surveillance of myocarditis and pericarditis following vaccination with COVID-19 mRNA vaccines in persons aged 12 to 39 years in Italy: A multi-database, self-controlled case series study
Source: PLoS Med. 2022 Jul 28;19(7):e1004056. doi: 10.1371/journal.pmed.1004056 (PMC9333264; doi:10.1371/journal.pmed.1004056)
Supplement: Attachment — Submitted filename: Italian paper review.docx [file pmed.1004056.s025.docx]

This potentially important paper on the issue of mRNA vaccines and myocarditis/pericarditis is based on a thorough analysis of a large Italian dataset. The paper is very clear; the methods are appropriate and well explained; the results are compelling; and possible shortcomings are well discussed in context. As a statistician, I focused mainly on methodological aspects. Overall, I thought this was an excellent paper and I strongly recommend publication.

The authors clearly have an excellent understanding of SCCS methodology and recent work on its application to COVID-19 vaccine safety. They are right to point out (lines 365 – 367) that unvaccinated cases should be included when the SCCS method adapted for event-dependent exposures is used. They explain that it was not possible to do so here. However, they argue that, in this application, this is likely to have little bearing on the results. I agree with them on this point, as a substantial number of events occurred before the first vaccination, and standard SCCS analyses starting at date of vaccination produce very similar results.

The other sensitivity analyses are well chosen and reinforce the results. The calculation of excess cases per 100,000 vaccinated also adds relevant context to the results.

I also very much liked their structured approach to hypothesis testing: focusing first on investigating the overall effect for both vaccines and all risk periods combined; and having established that there is an effect to be studied, then going on to tease it out in greater detail through subgroup analyses. This structured approach provides some insurance against multiple testing.

I have no major criticisms of the paper requiring a resubmission. I have only a few largely trivial points which could be fixed when the paper is prepared for publication.

1. Line 44 and line 178: “was fitted using a conditional Poisson regression model”: strictly speaking, this is not quite right. The SCCS model for event-dependent exposures is not likelihood-based, but is fitted using unbiased estimating equations. You could say “was fitted using unbiased estimating equations” or just leave this out.
2. Line 119: information on age (not an age).
3. Line 153: “representing 36% of the population”: of what population? Of Italy?
4. Line 186: “we used the SCCS standard”: perhaps rephrase as “we used the standard SCCS”?
5. Line 237: “following the first dose of mRNA-1273; following a second dose…”: for greater clarity, state what risk period the EC relates to here (I presume it’s the 0 – 7 day risk period).
6. Table 4: some of the event counts in this table are pretty small, and the 95% confidence intervals for the corresponding relative incidences are likely to be approximate. Perhaps some mention should be made of this somewhere.
7. Figures 1 and 2: the terms “Cominarty” and “Spikevax” are used, whereas in the paper the vaccines are referred to as BNT162b2 and mRNA-1273. It might be best to stick to the same terms, or introduce the labels in the paper.

In conclusion: I strongly recommend acceptance of this paper, without revision. While the authors might choose to address the very minor points I raise, I think the paper is publishable in its current form.

Paddy Farrington

Professor emeritus of statistics, The Open University
