# Supplementary material for: Postmarketing active surveillance of myocarditis and pericarditis following vaccination with COVID-19 mRNA vaccines in persons aged 12 to 39 years in Italy: A multi-database, self-controlled case series study
Source: PLoS Med. 2022 Jul 28;19(7):e1004056. doi: 10.1371/journal.pmed.1004056 (PMC9333264; doi:10.1371/journal.pmed.1004056)
Supplement: Attachment — Submitted filename: Massari_ResponseReviewer PLoSM (26.05.2022).docx [file pmed.1004056.s026.docx]

**RESPONSE TO REVIEWERS**

**Post-marketing active surveillance of myocarditis and pericarditis following vaccination with COVID-19 mRNA vaccines in persons aged 12-39 years in Italy: a multi-database, self-controlled case series study**

Massari M & Spila Alegiani S et al.

**REQUESTS FROM THE EDITORS:**

*Authors’ reply: Dear Editor, thank you for your recommendations, observations and suggestions. Below we report our point by point answers (number of pages and lines referred to the clean version of the paper).*

**(1)**

In addition, we request that you upload any figures associated with your paper as individual TIF or EPS files with 300dpi resolution at resubmission; please read our figure guidelines for more information on our requirements: <http://journals.plos.org/plosmedicine/s/figures>. While revising your submission, please upload your figure files to the PACE digital diagnostic tool, <https://pacev2.apexcovantage.com/>. PACE helps ensure that figures meet PLOS requirements. To use PACE, you must first register as a user. Then, login and navigate to the UPLOAD tab, where you will find detailed instructions on how to use the tool. If you encounter any issues or have any questions when using PACE, please email us at [PLOSMedicine@plos.org](mailto:PLOSMedicine@plos.org).

*Authors’ reply:* We uploaded figures in the format requested and we uploaded the figures to the PACE digital diagnostic tool as requested

**(2)**

The Data Availability Statement (DAS) requires revision. If the data are not freely available, please describe briefly the ethical, legal, or contractual restriction that prevents you from sharing it. Please also include an appropriate contact (web or email address) for inquiries (this cannot be a study author).

*Authors’ reply: we have reformulated the Data Availability statement as following:*

*“Data can not be shared publicly under article 9 of regulation (EU) 2016/679. Data are available from the Data Protection Officer of Istituto Superiore di Sanità, Doctor Carlo Villanacci, e-mail* [*responsabile.protezionedati@iss.it*](mailto:responsabile.protezionedati@iss.it)*, for researchers who meet the criteria for access to confidential data”.*

**(3)**

Throughout, please consider using an alternative term instead of ‘brand’ to reflect the non-proprietary presentation of the vaccines e.g. product/type.

*Authors’ reply: we have substituted “brand” with “product” throughout the text.*

Abstract Methods and Findings:

**(4)**

* Please ensure that all numbers presented in the abstract are present and identical to numbers presented in the main manuscript text.

*Authors’ reply: we have corrected them.*

**(5)**

* Please include the important dependent variables that are adjusted for in the analyses.

*Authors’ reply: we added it at lines 45-46, page 2.*

**(6)**

* In the last sentence of the Abstract Methods and Findings section, please describe the main limitation(s) of the study's methodology.

*Authors’ reply: A sentence about main limitations was added at lines 59-61, page 3.*

**(7)**

Citations should be in square brackets and precede punctuation throughout.

*Authors’ reply: we have changed the reference citations throughout the text.*

**(8)**

Line 105: Please include ‘to our knowledge’ to temper claims of primacy.

*Authors’ reply: It was added at line 105, page 5.*

**(9)**

The terms gender and sex are not interchangeable (as discussed in <http://www.who.int/gender/whatisgender/en/> ); please use the appropriate term.

*Authors’ reply: we substituted the term “gender” with “sex” throughout the text*

**(10)**

Please confirm whether informed consent from participants was written or verbal.

*Authors’ reply: the informed consent was not collected from participants since the study used routinely collected data from health care registries.*

**(11)**

I could not locate the RECORD-PE checklist? Please check this was included and cite in your Methods (S1 Checklist).

*Authors’ reply: we added a sentence in the Methods section at line 204, page 9.*

**(12)**

When completing the checklist, please use section and paragraph numbers, rather than page numbers.

*Authors’ reply: we added section, paragraph, line and page number as requested.*

**(13)**

"Did your study have a prospective protocol or analysis plan? Please state this (either way) early in the Methods section.

a) If a prospective analysis plan (from your funding proposal, IRB or other ethics committee submission, study protocol, or other planning document written before analyzing the data) was used in designing the study, please include the relevant prospectively written document with your revised manuscript as a Supporting Information file to be published alongside your study, and cite it in the Methods section. A legend for this file should be included at the end of your manuscript.

b) If no such document exists, please make sure that the Methods section transparently describes when analyses were planned, and when/why any data-driven changes to analyses took place.

*Authors’ reply: a prospective protocol (in Italian language) was submitted to the Italian ethical committee, and almost all the key elements of the SCCS method were described. The only decision not pre-specified in the protocol was the exposure risk period (21 days, further subdivided into 3 equal interval of seven days) that was decided before data collection and analysis and to take into account the interval between the vaccine dose administrations (immunization schedule: 21 days interval for BN162b2 and 28 days interval for mRNA-1273). A sentence was added in the method section (lines 173-175, page 8).*

c) In either case, changes in the analysis-- including those made in response to peer review comments-- should be identified as such in the Methods section of the paper, with rationale.

*Authors’ reply: no substantial changes in the analyses were made, we added, on request, additional sensitivity analyses that are reported in the supplementary material and described in the method section. We added a sentence in the Method section (lines 189-190 and 195, page 8; lines 200-201, pages 8-9).*

**(14)**

Please include the unadjusted comparisons as well as the adjusted comparisons in Tables 3 and 4 (if space is an issue, the unadjusted comparisons can be placed in the Supporting Information and cited in the Results).

*Authors’ reply: Unadjusted comparisons were reported in the supplementary material (S4 Table and S5 Table) and cited in the Results section (lines 236-237, page 12 and line 256, page 13). In the headings of Tables 2 and 3 and Table S6-S15 the word “adjusted” is now referred to Relative Incidence. In Table 3, Relative Incidence for females 12-39 years vaccinated with mRNA-1273 vaccine was unadjusted for unstable estimates of the seasonal effect due to the small number of cases. This is now specified within the table with the following note: “*** unadjusted relative incidences due to the small number of cases”*

**(15)**

References: Please use et al only after listing the first six

*Authors’ reply: we added the missing authors before et al.*

**(16)**

Please update figures to use non-proprietary rather than commercial vaccine names, for consistency with the main text.

*Authors’ reply: we apologize for the typo, we have substituted “Comirnaty” with “BN162b2” and “Spikevax” with “mRNA-1273” in Figure 1 and Figure 2.*

**COMMENTS FROM THE REVIEWERS:**

**Reviewer #1:**

This paper presents the analysis of routinely collected data on speciﬁc side eﬀects of mRNA vaccines in young people in Italy.

I was asked for a statistical report and I interpret that to include all aspects of the design and conduct of the study.

*Authors’ reply: Thank you for your comments, observations and suggestions. Below we report our point by point answers (number of pages and lines referred to the clean version of the paper).*

**(17)**

Page 7 I wonder whether the fact that all four regions are in the north of Italy is worth mentioning. I have no idea about north–south health gradients in Italy but I imagine there must be some.

*Authors’ reply: we have specified in the text that three regions (Friuli Venezia Giulia, Lombardia and Veneto) are in the northern Italy and one region, Lazio, in the central of Italy. Vaccination campaign as well as accuracy of vaccination registry across all regions in Italy was homogeneous so we do not expect that the inclusion of regions from the south of Italy would be likely to affect the results. We added a sentence at line 157, page 7.*

**(18)**

Page 7 I think in the results we should be told how many people had to be excluded for missing sex, age, or vaccine details. I imagine this was quite rare but unless we know we cannot say whether any extra analysis is needed to cope with missingness.

*Authors’ reply: The Italian regional healthcare databases used for the SCCS analyses (population registry, hospital discharge, emergency care visit and vaccination registries) are generally complete and of high quality because they are collected for administrative purposes. [Trifirò G, Gini R, Barone-Adesi F, et al. The role of European Healthcare Databases for post-marketing drug effectiveness, safety and value evaluation: where does Italy stand? Drug Saf 2019;42:347-63. Skrami E, Carle F, Villani S, et al. Availability of Real-World Data in Italy: A Tool to Navigate Regional Healthcare Utilization Databases. Int J Environ Res Public Health. 2019;17(1):8. Published 2019 Dec 18. doi:10.3390/ijerph17010008].*

*We had no missing observations for age and sex since they are key information for the population registry and they are collected comprehensively and we had a very low proportion of missing or inconsistent observations (0.7%) for vaccines so we are not concerned that our analysis would be biased. A flow chart (S3 figure) was uploaded in the supplementary material and this information was added in the result section (lines 215-216, page 9). We have also rephrased in the method section the sentence regarding missing data (lines 162-163, page 7).*

**(19)**

Page 13 on onwards The authors deﬁned the period of interest as 0-21 days (see page 7) but their results and analysis focus on the three separate time periods. I can see that the hypothesis may have been that early eﬀects were to be expected or that the patho–physiology demands the sub–division but why not present the 0–21 results? After all as a patient I want to know if I am going to experience a side eﬀect and am not really interested in whether it occurs on day 5, 10 or 15.

*Authors’ reply: All the reported analyses are performed using the 0-21 day risk period, further subdivided into 3 pre-specified time-periods: [0-7), [7-14), and [14-21). Therefore, the unexposed baseline period (reference period) is the same for all the analyses made, except as specified in the sensitivity analyses. To clarify it, we added in the text (lines 237-240, page 9) and in table 2, the results for the entire risk period 0-21 days.*

**(20)**

Page 14 Perhaps have another row with the overall totals (346 and 95)? It took me a moment to work out why 11 + 23 + 3 + 1 + 2 6= 55

*Authors’ reply: the total number of cases for each vaccine product is reported in the corresponding columns headers (n. 346 for BNT162b2 and n. 95 for mRNA-1273). N.55 and n. 272 are the number of cases in the reference period that summed up to the cases in the risk intervals [0-7) [7-14) [14-21) yield the total number of cases (346 and 95). In reviewing the Table we realized that there was a mistake about the BNT162b2 cases in the reference period: there are 272 cases not 172. We corrected the data in Table 2.*

**(21)**

Page 14 The subset analyses were pre–speciﬁed but presenting them does lead the reader to assume that the diﬀerences between men and women and between age groups are substantial. Was any form of formal interaction test carried out? If not then I think it would be a helpful addition.

*Authors’ reply:* *In the standard SCCS model, interaction may be formally investigated by ﬁtting the interaction term between sex/age and exposure using a likelihood ratio test. In the SCCS extension for event-dependent exposures (that uses unbiased estimating equations), this is not possible as no likelihood has been defined; it is possible to estimate separate effects by using subgroups analysis with confidence intervals that are mostly overlapping as described in S4 figure.*

**(22)**

Page 19 Would it be helpful to tabulate the results of the previous studies in some way to ease comparison of the totality of global evidence?

*Authors’ reply: we have produced a table of the previous studies we considered at the time we conceived our study and included as the supplementary material (S1 Table).*

**(23)**

Page 26 I do not think elaborate is quite the right word here. As an adjective it usually means complicated. Perhaps produced or compiled might be better.

*Authors’ reply: we substituted the term “elaborate” with “process” (S1 Fig) and “elaborating process” with “Flow Diagram” at line 156, page 7.*

**(24)**

Figures Suddenly introducing the trade names seems strange. Perhap cross–reference them to the names used in the main text? Articles about vaccination may attract lay readers to an open access journal and, at least in my country, the lay public will not be familiar with them. In fact I think the only place I have seen Comirnaty is on my vaccine pass (which incidentally tells me it is manufactured by Biontech just to confuse matters further). I did not know the name Spikevax at all.

*Authors’ reply: we apologize for the typo, we substituted “Comirnaty” with “BN162b2” and “Spikevax” with “mRNA-1273” in Figure 1 and Figure 2.*

Points of more substance

**(25)**

There is much vaccine hesitancy in the world and this article has a focus, as planned, on side eﬀects. The authors do refer to the balance between side eﬀects and beneﬁts but I feel that they rather understate this. I know their research has not studied the beneﬁts but a slightly stronger comment on beneﬁts versus side eﬀects would not be out of place.

*Authors’ reply: We agree with the reviewer that vaccine hesitancy is a major issue worldwide, particularly in the context of COVID-19 pandemic and we understand the reviewer’s concern. We have reflected on this comment and we modified the sentence in the Conclusions section at lines 405-407, page 20.*

Summary

No major issues.

Michael Dewey

**Reviewer #2:**

Myocarditis/pericarditis in relation to mRNA vaccinations has been a topic of interest lately due to ongoing concerns of an association. This study, along with others based in different countries, found significant associations between mRNA vaccinations and this event. This paper in particular finds an association [0-7) days after vaccination for mRNA-1273 following both doses and BNT162b2 following the second dose. This study is of interest as it supports findings found in other countries but uses self-controlled case series adapted to handle event-dependent exposures. This is a well-written study, but I have a few suggestions that I think would improve the paper and that I ask the authors to consider:

*Authors’ reply: Thank you for your comments, observations and suggestions. Below we report our point by point answers (number of pages and lines referred to the clean version of the paper).*

**(26)**

1. An important assumption in this study is that there are event-dependent exposures. The authors mention that they conduct a subanalysis using SCCS standard, starting the observation time at the first and second dose. I assume that this means that all time before vaccination is not considered in the SCCS analysis, and the days following the 21-day risk period are the reference period.

*Authors’ reply: Yes, we confirm your observation.*

While I agree that there could be bias introduced due to a delayed vaccination following an event, I do not think that removing time prior to vaccination in the standard SCCS method and comparing it to your main results is a sufficient example in itself of why the modified SCCS model should be used. I would recommend doing a plot similar to Figure 1, but include time before the first vaccination as well so that it can be investigated whether there is a visible "dip" in events just prior to vaccination. If so, introducing a pre-risk period is justified.

*Authors’ reply: Yes, Figure 1 is now modified in the manuscript, showing clearly the existence of a “dip” prior to vaccination. Introducing a pre-risk period in the standard method is only recommended if the event-dependence on exposure is relatively short and well defined which may not be the case.[Ghebremichael-Weldeselassie Y, Jabagi MJ, Botton J, Bertrand M, Baricault B, Drouin J, Weill A, Zureik M, Dray-Spira R, Farrington P. A modified self-controlled case series method for event-dependent exposures and high event-related mortality, with application to COVID-19 vaccine safety. Stat Med. 2022 May 10;41(10):1735-1750. doi: 10.1002/sim.9325. Epub 2022 Jan 28. PMID: 35092037]*

Long event delays or preclusions of the exposure will not be visible on this plot, but doing the standard SCCS analysis without the exclusion of this time as a sort of sensitivity analysis to see whether the results are heavily biased by the event-dependent exposure assumption could aid in this investigation. If the event delays or precludes the exposure, then the relative incidence will be biased upwards. Alternatively, (Farrington CP, Whitaker H, Weldeselassie YG. Self-Controlled Case Series Studies. A Modelling Guide with R. CRC Press, 2018) recommends removing post-event exposures in a standard SCCS analysis in order to display this bias (pg. 237-239).

*Authors’ reply: We have performed all the analyses recommended by the referee. They all resulted in an overestimation to various extents (especially in mRNA-1273) when compared to the primary analysis based on event-dependent modified SCCS method. As expected, the only exception is for the analysis where the standard SCCS is applied with the “trick” of beginning observation at exposure. This is reassuring on the choice of the modified SCCS method. The results of the new sensitivity analyses applying the standard SCCS are now shown in the supplemental material (S17 Table) and commented in the results section of the paper at lines 302-304, page 16.*

**(27)**

2. I do not follow the conclusion "According to our results the risk increased after the second dose and in the youngest for both vaccines, remained moderate following vaccination with BNT162b2, while was higher in males following vaccination with mRNA-1273.”

a. The youngest age group (12-17) did not have any estimates for mRNA-1273, so how can the risk be increased in the youngest for both vaccines?

*Author’s reply: We rephrased the Conclusion section (see the answer below at point c)*

b. I would also use the phrase "was not statistically significant" instead of "moderate" when discussing RI.

*Author’s reply: We rephrased the Conclusion section (see the answer below at point c)*

c. In general, this sentence is a bit hard to follow. Consider breaking it up into two.

*Author’s reply: we tried to better clarify the sentence in the Conclusion section at lines 402-404, page 20.*

**(28)**

3. As you reference later in the paper, different countries found varying results per dose when receiving the different vaccine brands. Therefore, I would be cautious assuming that heterologous vaccinations essentially do not exist in the primary analysis. Is this a reasonable assumption to make following the Italian vaccination plan that many do not get cross-vaccinated? I would report the results of the analysis where these individuals have been censored when they received a different vaccine brand as the main analysis to avoid the effect mixing the brands would have.

*Author’s reply: Among the sensitivity analyses we have conducted one excluding individuals who received two different vaccine products at the first and second dose, and another one censoring individuals at the time of second dose who received a different vaccine product (supplementary table S16). During the study period, among the 441 cases 18-39 years included in the analysis, only one subject received different product at dose 1 and dose 2 (first dose* mRNA-1273 *and second dose* BNT162b2*), thus both sensitivity analyses estimated same results of the primary analysis.*

**(29)**

4. This comment is regarding Table S12: Sensitivity Analysis: c) Heterologous vaccination (II). If it is correct that only one person's time is censored here (and it is a person who got an mRNA-1273 to BNT162b2 combination) and the rest of the analysis remains the same, why does this change the number of cases in BNT162b2 such that it is no longer estimable? Should it not be the same number of cases? Please recheck this analysis or explain the analysis in more detail.

*Author’s reply: Yes, it is correct. The number of cases and the estimates remain the same for BNT162b2. We apologize, while formatting the table we confused the double quote symbol ["] which means "as above" with the symbol *** (Considering the small number of cases, it was not possible to provide any estimate). We corrected it (Table S16).*

**(30)**

5. Using the time after the exposure period for a reference period allows for more cases and power to your study instead of just using the time prior to vaccination as the reference period.

*Author’s reply: The reference period was defined as any time of observation out of the risk period i.e. time before, after and between dose administration. Please see the S2 Figure schematic presentation of the SCCS method as you suggested.*

However, performing a sensitivity analysis on the length of the exposure period is, therefore, more important as it affects your reference period. For that I have two comments:

a. Have you preformed any sensitivity analyses using this risk period (either shortening or extending the length) justifying your choice of risk period? Other countries listed in your discussion have a risk period of 28-days.

*Author’s reply: the 0-21 days risk period was pre-specified before data collection and analysis. Considering the schedule of mRNA vaccination in Italy (21 day for BNT1622b and 28 days for mRNA-1273) we decided in the primary analysis to not extend the risk period over 21 days to avoid overlapping between doses.*

b. I would recommend adding a sensitivity analysis either where you increase the number of days in your risk period or exclude some time shortly following your risk period so that you can investigate whether your unexposed period remains unbiased from potential delayed reported events related to your exposure.

*Author’s reply: as you suggested, we added two new sensitivity analyses extending and reducing the overall risk period to 0-28 and 0-14 days. These sensitivities were added in S16 Table and we also added a sentence on this in the Method section at line 195, page 8.*

**(31)**

6. A schematic illustration of the time windows used in the SCCS analysis would benefit the reader. This could potentially be put in as a supplementary plot and referenced to in "Statistical analysis" or "Definition of exposures".

*Author’s reply: we agree with the reviewer and we added a new figure in the supplementary material (S2 Figure) cited in “Definition of exposure” at line 178, page 8.*

Minor comments:

**(32)**

* Line 186: SCSS -> SCCS

*Authors’ reply: we rephrased the sentence and corrected the acronym lines 200-201, pages 8-9.*

**(33)**

* Table 1: Please format the table so that the groupings of characteristics are more visible and the group-wise percentages are easier to see.

*The author’s reply: the table has now been formatted as suggested. We moved Table 1 in the Supplementary material as requested by another reviewer (S2 Table). We added a new sentence where the S2 Table was cited (lines 217-218, page 9)*

**(34)**

* Fig 1: Please change the heading of the figure to BNT162b2 and mRNA-1273 to match the rest of the paper.

*Authors’ reply: we apologize for the typo, we have substituted “Comirnaty” with “BNT162b2” and “Spikevax” with “mRNA-1273” in Figure 1 and Figure 2.*

**(35)**

* Lines 317-318: The statement in these lines is correct for mRNA-1273, but only after the second dose for Pfizer. Consider rephrasing.

*Authors’ reply: we rephrased as suggested at line 328, page 17.*

**(36)**

* Line 338: "also reported an higher risk in male" -> "also reported a higher risk in males"

*Authors’ reply: this has now been corrected as suggested at line 351, page 17.*

**(37)**

* Lines 355-356: This sentence does not make sense. Consider rephrasing. "The large sample size (about 3 million vaccinated people aged 12-39 years) allowed us to look at fine risk intervals following vaccination and conduct several subgroup analyses."

*Authors’ reply: the statement was rephrased as suggested at lines 370-371, page 19.*

**(38)**

* Lines 380-383: This sentence does not make sense. Consider rephrasing.

*Authors’ reply: We recognise that it was not clear and we decided to remove it from the Conclusion section.*

**(39)**

* Line 389: Remove "s" in "conclusions".

*Authors’ reply: this has now been corrected as suggested at line 401, page 20.*

**Reviewer #3:**

Massari et al. estimate the increased risk for myocarditis and pericarditis following receipt of mRNA Covid-19 vaccines. The study is interesting, but I do have concerns that merit addressing.

*Authors’ reply: Thank you for your comments, observations and suggestions. Below we report our point by point answers (number of pages and lines referred to the clean version of the paper).*

Note: The system describes this as a revision. I was not involved in the original submission and know nothing of previous comments. I therefore review this as I would review the original submission.

== Major ==

**(40)**

More needs to be said about the quality of the data, particularly as it synthesizes various sources. Are all sources guaranteed to be complete? If not, why and how incomplete?

*The Italian regional healthcare databases used for the SCCS analyses (population registry, hospital discharge, emergency care visit and vaccination registries) are generally complete and of high quality because they are collected for administrative purposes. [Trifirò G, Gini R, Barone-Adesi F, et al. The role of European Healthcare Databases for post-marketing drug effectiveness, safety and value evaluation: where does Italy stand? Drug Saf 2019;42:347-63. Skrami E, Carle F, Villani S, et al. Availability of Real-World Data in Italy: A Tool to Navigate Regional Healthcare Utilization Databases. Int J Environ Res Public Health. 2019;17(1):8. Published 2019 Dec 18. doi:10.3390/ijerph17010008]. A flow chart (S3 figure) was uploaded in the supplementary material and this information was added in the result section (lines 215-216, page 9). We have also rephrased in the method section the sentence regarding missing data (lines 162-163, page 7).*

**(41)**

How long is the history for each person.?How complete is the follow-up? Etc.

*Each subject was observed from the beginning of the study (27/12/2020) to the end of follow up (latest date for which data on outcome are available or death). In the text of the manuscript we reported that the median follow-up time of vaccinated persons. We think it would be informative to add also the follow up time after the event. We included this information at line 225, page 10.*

**(42)**

In the introduction, the authors should explicitly state the scientific gap they aim to feel. From what I understand, the relative risk has already been quantified for these two vaccines, including by age group and sex. What then is the goal of the study?

*Authors’ reply: at the end of the Introduction section, a paragraph is now included with considerations on the gaps we aimed to fill and the goal of the study is better defined at lines 105-111, page 5.*

**(43)**

I do not have intimate knowledge of the SCCS methodology, and particularly not of its extension to situations in which exposure depends on past events (like it does here, as past myocarditis lowers the probability of vaccination). This is a critical aspect of this study, and others more knowledgeable should be sought to review it, as many questions arise including issues of confounding by calendar time, inclusion only of vaccinated (e.g., why isn't information on the unvaccinated used? Does this not result in bias?), etc.

*Authors’ reply: We thank the reviewer of this important observation on the SCCS methodology. The problem of including only vaccinated individuals is an important one, but the effect of this choice depends on numbers. We cite directly one of the reviewers (Paddy Farrington, the creator of the SCCS methodology): “They are right to point out (lines 365 – 367) that unvaccinated cases should be included when the SCCS method adapted for event-dependent exposures is used. They explain that it was not possible to do so here. However, they argue that, in this application, this is likely to have little bearing on the results. I agree with them on this point, as a substantial number of events occurred before the first vaccination, and standard SCCS analyses starting at date of vaccination produce very similar results”*.

**(44)**

In the same context, While I hesitate to suggest a different analysis and I understand the within-person advantage of SCCS, but considering that other than previous myocarditis, there aren't known strong confounders for the vaccination => myocarditis effect, why not use a standard cohort design with a modified Poisson model? This is even more warranted when considering that the authors acknowledge that not including unvaccinated persons could lead to bias.

*Authors’ reply: We believe that SCCS is a key methodology for studying the safety of vaccines (Weldeselassie YG, Whitaker HJ, Farrington CP. Use of the self-controlled case-series method in vaccine safety studies: review and recommendations for best practice. Epidemiol Infect 2011;139 (12):1805–17). The SCCS design has emerged as a key methodology for studying the safety of vaccines and pharmaceutical drugs. It is a design particularly suitable in the research of adverse events of vaccines where there could be differences between vaccinated and unvaccinated persons and where it can be difficult to find a suitable comparison group since most of the population has received the vaccine, which is the case in Italy, where unvaccinated patients may be different from vaccinated ones, also for factors that are not measurable or known by linking our health care databases as smoking, body weight, socioeconomic factors, and lifestyle. [Ghebremichael-Weldeselassie Y, Jabagi MJ, Botton J, Bertrand M, Baricault B, Drouin J, et al. A modified self-controlled case series method for event-dependent exposures and high event-related mortality, with application to COVID-19 vaccine safety]. Moreover, we used the SCCS design because by using a different design we could reinforce evidence about COVID-19 vaccine adverse effects when comparing our results with those of the other studies with different study design.*

**(45)**

The authors should avoid language that stems from statistical significance. E.g., saying that no association was found between the first 7 days after the first dose of the Pfizer vaccine and the outcome or that "increased risk was present only in…". The reason is simple - if statistical significance is evoked, then all its tenets must be adhered to, including adjusting for multiple comparisons, which was not performed here. A simple reporting of the CI, then, would suffice.

*Authors’ reply: We modified the manuscript in order to avoid explicit reference to significance. We added a new sentence in the Method section at lines 184-186, page 8.*

== Minor ==

**(46)**

The abstract result section is overly detailed. Should only be overall effects per each vaccine and maybe some specific interesting subgroup results.

*Authors’ reply: The result section of the abstract was synthesized (lines 46-59, pages 2-3).*

**(47)**

The conclusion in the abstract "This population-based study suggested that mRNA vaccines were associated with myocarditis/pericarditis in the population younger than 40 years, whereas no association was observed in older subjects" is weird, considering that this study is limited to ages 12-39. Later I see that this was a secondary analysis. Its results then, should not appear in the abstract.

*Authors’ reply: The sentence “whereas no association was observed in older subjects” has been removed as suggested.*

**(48)**

Persons with missing data were excluded. Why? How many were there? A population flow chart should be included and the number of missing mentioned.

*Authors’ reply: In our study, we had a very low proportion of missing or inconsistent observations (0.7%) for vaccines so we are not concerned that our analysis would be biased. Data was shown in S3 figure, uploaded in the supplementary material, and we also added this information in the Methods (lines 162-164, page 3) and Result sections (line 215-216, page 9).*

**(49)**

The authors dedicate Table 1 to all those vaccinated with mRNA vaccines. This is not warranted. The study population in a SCCS design are those that experienced an event and were included. The complete population should only be mentioned in the context of the population flow chart (which I do not see in this submission). Similarly, mentioning the full population as a strength does not seem to make sense.

*Authors’ reply: we removed the table from the manuscript. However, for completeness of information, we moved Table 1 in the supplementary material as S2 Table. We also added a flowchart to describe the population selection in the supplementary material (S3 Fig).*

**(50)**

The structure of Table 2 is not optimal. The stratification by age makes the table hard to understand and does not seem helpful.

*Authors’ reply: Table 2 (now renamed Table 1) was simplified.*

**(51)**

The effect estimate of interest is called "relative incidence" throughout. It would be preferable to use the more informative and accurate "incidence rate ratio".

*Authors’ reply: In the papers using the SCCS method, as well in the book by P. Farrington (Farrington CP, Whitaker H, Weldeselassie YG. Self-Controlled Case Series Studies. A Modelling Guide with R. CRC Press, 2018), the effect estimate is called “Relative incidence”, thus we decided to use this term for consistency with SCCS methodology.*

**(52)**

The strengths section is better removed. The merit of a study should be evident from the reporting and not specifically mentioned. Moreover, most of this section is devoted to what is actually a limitation - the exclusion of unvaccinated individuals.

*Authors’ reply: We preferred not to remove the paragraph about strengths of the study, since there could be a part of the audience not particularly confident in critically appraising epidemiological studies who might find useful to read these few sentences on strengths alongside with those about the study limits, particularly in the case of the SCCS and multidatabase studies.*

**Reviewer #4:**

This potentially important paper on the issue of mRNA vaccines and myocarditis/pericarditis is based on a thorough analysis of a large Italian dataset. The paper is very clear; the methods are appropriate and well explained; the results are compelling; and possible shortcomings are well discussed in context. As a statistician, I focused mainly on methodological aspects. Overall, I thought this was an excellent paper and I strongly recommend publication.

The authors clearly have an excellent understanding of SCCS methodology and recent work on its application to COVID-19 vaccine safety. They are right to point out (lines 365 – 367) that unvaccinated cases should be included when the SCCS method adapted for event-dependent exposures is used. They explain that it was not possible to do so here. However, they argue that, in this application, this is likely to have little bearing on the results. I agree with them on this point, as a substantial number of events occurred before the first vaccination, and standard SCCS analyses starting at date of vaccination produce very similar results.

The other sensitivity analyses are well chosen and reinforce the results. The calculation of excess cases per 100,000 vaccinated also adds relevant context to the results.

I also very much liked their structured approach to hypothesis testing: focusing first on investigating the overall effect for both vaccines and all risk periods combined; and having established that there is an effect to be studied, then going on to tease it out in greater detail through subgroup analyses. This structured approach provides some insurance against multiple testing.

I have no major criticisms of the paper requiring a resubmission. I have only a few largely trivial points which could be fixed when the paper is prepared for publication.

*Authors’ reply: Thank you for your comments, observations and suggestions. Below we report our point by point answers (number of pages and lines referred to the clean version of the paper).*

**(53)**

1. Line 44 and line 178: “was fitted using a conditional Poisson regression model”: strictly speaking, this is not quite right. The SCCS model for event-dependent exposures is not likelihood-based, but is fitted using unbiased estimating equations. You could say “was fitted using unbiased estimating equations” or just leave this out.

*Authors’ reply: We modified the text in the abstract at line 44, page 2 and in the method section at line 183, page 8.*

**(54)**

2. Line 119: information on age (not an age).

*Authors’ reply: This has now been corrected as suggested at line 123, page 6.*

**(55)**

3. Line 153: “representing 36% of the population”: of what population? Of Italy?

*Authors’ reply: we clarified it at line 157-158, page 7.*

**(56)**

4. Line 186: “we used the SCCS standard”: perhaps rephrase as “we used the standard SCCS”?

*Authors’ reply: This has now been corrected as suggested throughout the text.*

**(57)**

5. Line 237: “following the first dose of mRNA-1273; following a second dose…”: for greater clarity, state what risk period the EC relates to here (I presume it’s the 0 – 7 day risk period).

*Authors’ reply: we clarified the risk period window at lines 247-248, page 12.*

**(58)**

6. Table 4: some of the event counts in this table are pretty small, and the 95% confidence intervals for the corresponding relative incidences are likely to be approximate. Perhaps some mention should be made of this somewhere.

*Authors’ reply: we have realized that obtaining confidence intervals for EC should take into account the fact that the corresponding Relative Incidence and number of events are correlated. Therefore, we recalculated the 95% Confidence Intervals using non-parametric bootstrapping (10,000 replications) for RIs and ECs. The 95% CI of RI were quite consistent with those estimated by the model. We decided to update the 95% CI of EC (now wider than previous) in the text (lines 189-190, page 8) and in tables 2 and 3 of the manuscript and S6-S15 of the Supplementary material.*

**(59)**

7. Figures 1 and 2: the terms “Cominarty” and “Spikevax” are used, whereas in the paper the vaccines are referred to as BNT162b2 and mRNA-1273. It might be best to stick to the same terms, or introduce the labels in the paper.

*Authors’ reply: we apologize for the typo, we substituted “Comirnaty” with “BNT162b2” and “Spikevax” with “mRNA-1273” in Figure 1 and Figure 2.*

In conclusion: I strongly recommend acceptance of this paper, without revision. While the authors might choose to address the very minor points I raise, I think the paper is publishable in its current form.

Paddy Farrington

Professor emeritus of statistics, The Open University

**AUTHORS’ CORRECTIONS:**

a) *We made a correction of follow-up time in the Results section at line 216, page 9.*
b) *In Table 2 (ex Table 3), the number of events in the reference period has been corrected (n=272).*
c) *In Table 3 (ex Table 4), EC of mRNA-1273 vaccine, age 18-29 years is 3.4 at Dose 1 and 8.6 at Dose 2. We changed values also in the Results of the Abstract (line 58, page 3) and the main text at line 277-279, page 14).*
d) *We cited the study of Karlstad et al. (was published while our paper was under review) in the discussion since we considered it important to update the research on mRNA vaccines-myocarditis/pericarditis and we add a new sentence at lines 354-356, page 18.*
e) *We substituted “confounders” and/or “covariates” with “comorbidities” and/or “characteristics” throughout the text where appropriate.*
